# Sanitary Plumbing

**Published:** 1899-10

**Authors:** Ed. Braden

**Affiliations:** San Antonio, Texas


### Correspondence.

---

### Sanitary Plumbing.

---

SAN ANTONIO, TEXAS, Sept. 7, 1899.

*Editor Texas Medical Journal.*

I take the liberty of mailing you some plumbing literature. Although our city has an ordinance regulating plumbing and house drainage and inspection, the claim made by Mr. Hyde is only too true. There is only one solution in the matter of reducing the death rate of cities, and that is the honest enforcement of our Texas plumbing law which takes the question of health measures out of political influence, removes the appointment of the plumbing inspector out of politics and places it under the direct control of the city health board and board of plumbing examiners.

We have an efficient city health officer, but when he orders a connection made to the sewer system (which cost the city half a million dollars) to abate a nuisance within ten days, the party gets an extension from the mayor or city clerk for sixty to ninety days. If any one is taken before the recorder he is let off on the plea of "no money."

When the city accepted the sewer system a general demand was made to stop polluting our beautiful streams, etc. The papers urged the city authorities to compel all having private connections with the river and ditches or using cesspools, to connect on to the new city sewers (a public cesspool). This was O. K., but instead of our city first having a house to house inspection, many a residence is connected in which the plumbing fixtures are not even trapped, much less vented. In all such cases plumbing fixtures act as a vent to our public sewer. No wonder our death rate increases. It does not require much argument to convince the medical profession that a sewer system under these conditions becomes a menace to the health of any community.

What must be the condition of our Texas cities in the absence of any regulation or inspection whatever, when they require no qualification of a plumber?

If an examining board is necessary to qualify the doctor, lawyer, druggist, etc., how much more so in a trade-profession in which the health of the public is involved?

It should be a penitentiary offense for a man to masquerade as a plumber.

We quarantine against the invasion of epidemic from outside, yet the people submit to a constant invasion from within.

I am glad to note that the Western Texas Medical Association at their last monthly meeting endorsed the Texas plumbing law. Hope the Texas State Medical Association will do the same at their next annual convention, in Waco. The medical profession can do much toward creating a demand for the enforcement of this law.

If it is a good move to take the public school question out of politics, would it not be of vastly more importance to take the care of the people's health out of politics?

When the "prevention of disease" is placed under the control of the health boards, we can feel assured that politics will cut no figure.

Very respectfully,

Ed. Braden, Jr.

